# Unregulated introduced fish (*Perca fluviatilis* Linnaeus, 1758) is host to zoonotic parasites in a small Mediterranean island

**DOI:** 10.1007/s00436-024-08264-4

**Published:** 2024-06-20

**Authors:** Anaïs Esposito, Gaël P. J. Denys, Vincent Haÿ, Paul-Jean Agostini, Joséphine Foata, Yann Quilichini

**Affiliations:** 1 Unité Mixte de Recherche Sciences Pour L′Environnement (UMR 6134 CNRS – Université de Corse Pascal Paoli), 20250 Corte, France; 2 Unité d’Appui à la Recherche Patrimoine naturel – Centre d’expertise et de données (UMS 2006 OFB – CNRS – MNHN – IRD), Muséum National d’Histoire Naturelle, 36 Rue Geoffroy-Saint-Hilaire CP 41, 75005 Paris, France; 3 Unité Mixte de Recherche Biologie des organismes et écosystèmes aquatiques (BOREA – MNHN, CNRS, IRD, SU, UCN, UA), 57 Rue Cuvier CP26, 75005 Paris, France; 4 Fédération Départementale de Pêche de La Corse, 20090 Ajaccio, France

**Keywords:** *Perca fluviatilis*, Introduced fish, *Clinostomum complanatum*, *Eustrongylides* spp., Recreational angling, First occurrence

## Abstract

Introduced species have a major impact on freshwater ecosystems, particularly on islands. Numerous fish species have been introduced in Corsica (Mediterranean island, southern France) as part of planned programs or clandestinely. The introduction of non-native freshwater fish species can have a range of impacts on the recipient ecosystem, including through the co-introduction of its pathogens. A sample of introduced perch *Perca fluviatilis* Linnaeus, 1758 from the artificial reservoir of Padula was examined following a report of parasites by an angler. The analyses revealed the occurrence of *Eustrongylides* sp. (Nematoda) and *Clinostomum complanatum* (Digenea), two zoonotic parasites in *P. fluviatilis*. Both parasites are reported for the first time in France. *Eustrongylides* sp. and *C. complanatum* may have been introduced with their fish intermediate hosts or through their final bird hosts. The occurrence of the two parasites raises concerns from both a veterinary and human health perspective as they can use a wide range of amphibians as intermediate hosts and can be acquired in humans through the consumption of raw or undercooked fish.

**Supplementary Information:**

The online version contains supplementary material available at 10.1007/s00436-024-08264-4.

## Introduction

Southern and Western Europe are a hotspot for biological invasions, with more than a quarter of total fish diversity being non-native species (Leprieur et al. 2008). Corsica is a small island in the Northwestern Mediterranean off Southern France and Western Italy, home to a particular fish fauna composed of only four native species and naturally lacking the Cypriniformes reported from the Ibero-Franco-Italian region (Roule 1933; Changeux 1998). The fish diversity in Corsica has however been artificially increased by the man-mediated introductions of more than 20 non-native fish species, the majority of them for recreational fishing purposes (Roche and Mattei 1997; Roché 2001). The first introduced species was the mosquitofish *Gambusia holbrooki* Girard, 1859 in an attempt to control mosquitoes (malaria control). Then, the brook trout *Salvelinus fontinalis* (Mitchill, 1814) and domestic brown trout *Salmo trutta* Linnaeus, 1758 were introduced in several mountain lakes. Afterwards, several species, e.g., the rudd *Scardinius erythrophthalmus* (Linnaeus, 1758), the carp *Cyprinus carpio* Linnaeus, 1758, the pikeperch *Sander lucioperca* (Linnaeus, 1758), the tench *Tinca tinca* (Linnaeus, 1758), and the roach *Rutilus rutilus* (Linnaeus, 1758), were introduced into several artificial reservoirs and spread by anglers to rivers and others reservoirs (Roché and Mattei 1997; Roché 2001). The rainbow trout *Oncorhynchus mykiss* (Walbaum, 1792) is regularly released in reservoirs and lower watercourses. The study of parasites carried by non-native hosts is of great importance as they may infect native host species, a phenomenon termed parasite spillover, as well as being transmitted to humans in the case of zoonotic parasites (Daszak et al. 2000; Prenter et al. 2004; Chai et al. 2005; Lymbery et al. 2014; Chalkowski et al. 2018; Eiras et al. 2018).

The European perch *Perca fluviatilis* Linnaeus, 1758 was introduced in Corsica outside the framework of any planned program and from an unknown source. Its presence was first noted in 1984 during the first emptying of the Uspidali reservoir. The species was then transferred to three second-category reservoirs in the eastern plain of Corsica (namely Peri, Alzitone, and Tepe-Rosse) and now forms sustainable populations in artificial habitats, i.e., several reservoirs (the four aforementioned ones plus Codole, Tolla, and Padula) and disused gravel quarries (Roché and Mattei 1997; Roché 2001; Fleury and Le Mesle 2022). In such habitats, this species is caught by recreational anglers and regularly consumed. Recently, the occurrence of live parasites was reported by recreational anglers in freshly caught *P. fluviatilis* from the Padula reservoir. There is no record of transfer of *P. fluviatilis* to Padula reservoir and its introduction in this ecosystem is believed to be the work of individual recreational anglers. In this reservoir, *P. fluviatilis* is known to occur since at least 2020. Parasites of *P. fluviatilis* have never been examined in Corsica.

The aim of this study was to determine whether the European perch population of the Padula reservoir can host zoonotic parasites, using morphological and molecular data. Their origin and potential impact on local fauna and human health are discussed.

## Material and methods

**Fig. 1:**
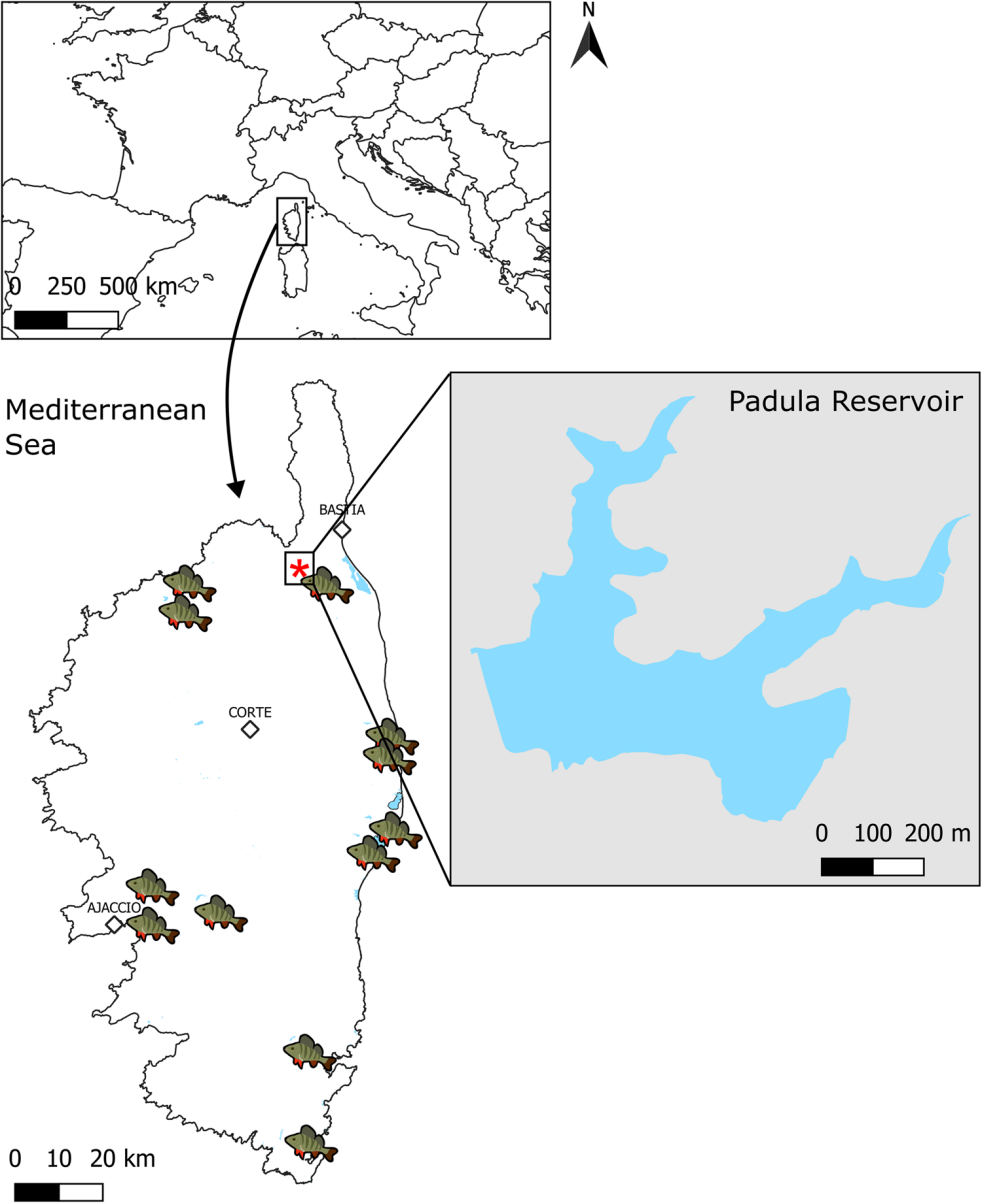
Localization of Corsica in the Mediterranean Sea and of the sampling site. Diamonds are the major cities in Corsica, the red asterisk is the sampling locality (Padula Reservoir), perch icons are the localities in which the occurrence of *Perca fluviatilis* has been reported according to Roché and Mattei (1997), Roché (2001), as well as Fleury and Le Mesle (2022)

Padula Reservoir (0.25 km^2^ surface area, 15 m maximum depth) (Fig. 1) is an artificial reservoir on the Furmicaiola stream, located in the north of the island at an altitude of 60 m above sea level, and administered by the *Office d’Equipement Hydraulique de Corse* (OEHC—Corsica water resources office). It was commissioned in 1992 and serves the purposes of irrigation and drinking water supply (Colonna 2021). To our knowledge, the fish population in Padula consists of *P. fluviatilis*, *S. lucioperca*, *C. carpio*, *S. erythrophthalmus*, the largemouth black bass *Micropterus salmoides* (Lacepède, 1802), and a few rainbow trout *O. mykiss*. Amphibians are also reported from the reservoir, including two endemic Urodela: the Corsican mountain newt *Euproctus montanus* (Savi, 1838) and the Corsican fire salamander *Salamandra corsica* Savi, 1838 according to the National Inventory of Natural Heritage (*Inventaire du Patrimoine Naturel* – INPN) database managed by the Muséum national d’Histoire naturelle (MNHN) and the French Agency of Biodiversity (OFB) (https://inpn.mnhn.fr/espece/cd_nom/59; https://inpn.mnhn.fr/espece/cd_nom/701817). Several water and piscivorous birds visit the reservoir, e.g., the grey heron *Ardea cinerea* Linnaeus, 1758, the little egret *Egretta garzetta* (Linnaeus, 1766), and the black-crowned black heron *Nycticorax nycticorax* (Linnaeus, 1758) (Table 1).

**Table 1:** Final bird host species reported for *Eustrongylides* spp. and *Clinostomum complanatum* from the literature, with information on their presence in Corsica. The presence of species is indicated according to the INPN database

Host species	Parasite species	Country	Reference	Presence in Padula Reservoir
Host-parasite records for *Clinostomum*				
Anseriformes				
Anatidae				
*Anas platyrhynchos* Linnaeus, 1758	*Clinostomum* sp.	Iran	(Gohardehi et al. 2013)	No, but reported from neighboring locality in Corsica
Charadriiformes				
Laridae				
*Larus argentatus* Pontoppidan, 1763	*C. complanatum*	-	(Gibson 2019)	No, but reported from neighboring locality in Corsica; Congenerics *Larus fuscus* and *Larus michahellis* reported from Padula Reservoir
*Larus canus* Linnaeus, 1758	*C. complanatum*	-	(Gibson 2019)	No, but present in the close-by Biguglia Lagoon
*Larus ridibundus Linnaeus, 1766*	*C. complanatum*	-	(Gibson 2019)	Yes
Ciconiiformes				
Ardeidae				
*Ardea alba* Linnaeus, 1758	*C. complanatum*	Australia	(Matthews and Cribb 1998)	No, but reported from neighboring locality in Corsica
	*C. complanatum*	Iran	(Shamsi et al. 2013)	
*Ardea cinerea* Linnaeus, 1758	*C. complanatum*	-	(Gibson 2019)	Yes
*Ardea purpurea* Linnaeus, 1766	*C. complanatum*	Iran	(Shamsi et al. 2013)	Yes
*Egretta garzetta* (Linnaeus, 1766)	*C. complanatum*	Australia	(Matthews and Cribb 1998)	Yes
	*C. complanatum*	Iran	(Shamsi et al. 2013)	
*Egretta novaehollandiae* (Latham, 1790)	*C. complanatum*	Australia	(Matthews and Cribb 1998)	Congeneric *Egretta garzetta* reported from Padula Reservoir
*Nycticorax caledonicus* (Gmelin, 1789))	*C. complanatum*	Australia	(Matthews and Cribb 1998)	Congeneric *Nycticorax nycticorax* reported from Padula Reservoir
*Nycticorax nycticorax* (Linnaeus, 1758)	*C. complanatum*	Iran	(Shamsi et al. 2013)	Yes
Pelecaniformes				
Phalacrocoracidae				
*Phalacrocorax carbo* (Linnaeus, 1758)	*C. complanatum*	Egypt	(El-Dakhly et al. 2018)	Yes
Host-parasite records for *Eustrongylides*				
Anseriformes				
Anatidae	*Eustrongylides mergorum*	Poland	(Kavetska et al. 2012)	Numerous Anatidae reported from Corsica e.g., *Anas platyrhynchos*, *Anas acuta, Mareca penelope*, *Netta rufina*, *Aythya ferina*
Ciconiiformes				
Ardeidae				
*Ardea alba* Linnaeus, 1758	*Eustrongylides ignotus*	Brazil	(Pinto et al. 2004)	No, but reported from neighboring locality in Corsica
	*Eustrongylides ignotus*	USA	(Locke 1961; Spalding and Forrester 1993)	
	*Eustrongylides* sp.	USA	(Caudill et al. 2014)	
	*Eustrongylides* sp.	USA	(Spalding 1990)	
	*Eustrongylides* sp.	USA	(Roffe 1988)	
*Ardea cocoi* Linnaeus, 1766	*Eustrongylides ignotus*	Brazil	(Pinto et al. 2004)	Congenerics *Ardea cinerea* and *Ardea purpurea* reported from Padula Reservoir
*Ardea herodias* Linnaeus, 1758	*Eustrongylides ignotus*	USA	(Bowdish 1948; Locke 1961; Spalding and Forrester 1993)	Congenerics *Ardea cinerea* and *Ardea purpurea* reported from Padula Reservoir
	*Eustrongylides* sp.	USA	(Spalding 1990)	
*Egretta caerulea* (Linnaeus, 1758)	*Eustrongylides* sp.	USA	(Spalding 1990)	Congeneric *Egretta garzetta* reported from Padula Reservoir
*Egretta thula* (Molina, 1782)	*Eustrongylides ignotus*	USA	(Spalding and Forrester 1993)	Congeneric *Egretta garzetta* reported from Padula Reservoir
	*Eustrongylides* sp.	USA	(Spalding 1990)	
*Egretta tricolor* (Müller, 1776)	*Eustrongylides* sp.	USA	(Spalding 1990)	Congeneric *Egretta garzetta* reported from Padula Reservoir
*Nycticorax nycticorax* (Linnaeus, 1758)	*Eustrongylides ignotus*	Brazil	(Pinto et al. 2004)	Yes
	*Eustrongylides* sp.	USA	(Caudill et al. 2014)	
Threskiornithidae				
*Platalea ajaja* Linnaeus, 1758	*Eustrongylides* sp.	USA	(Spalding 1990)	Congeneric *Platalea leucorodia* reported from neighboring locality
Pelecaniformes				
Anhingidae				
*Anhinga novaehollandiae* (Gould, 1847)	*Eustrongylides* sp.	Australia	(Sutherland et al. 2018)	No
Phalacrocoracidae				
*Phalacrocorax brasilianus* (Gmelin, 1789)	*Eustrongylides* spp.	Brazil	(Monteiro et al. 2011)	Congeneric *Phalacrocorax carbo* reported from Padula Reservoir
*Phalacrocorax carbo* (Linnaeus, 1758)	*Eustrongylides excisus*	Italy	(Rusconi et al. 2022)	Yes
	*Eustrongylides tubifex*	Japan	(El-Dakhly et al. 2012)	
	*Eustrongylides* spp.	Lithuania	(Švažas et al. 2011)	
*Phalacrocorax sulcirostris* (Brandt, 1837)	*Eustrongylides excisus*	Australia	(Shamsi et al. 2023a, b)	Congeneric *Phalacrocorax carbo* reported from Padula Reservoir

Ten specimens of *P. fluviatilis* (total length 208 ± 36 (116–248 mm), total weight 123 ± 47 (16–172 g)) were sampled by a recreational angler on 07/06/2023. Fish were brought back dead to the laboratory in an insulated bag by the angler, through the Angling Federation of Corsica (FDAAPPMA2). Fish were dissected and the body surface, opercular cavity, abdominal cavity, liver, swimbladder, and muscle tissue were thoroughly examined. However, neither the digestion (McGladdery 1986) nor the incubation (Shamsi and Suthar 2016) methods were used. As a result, an eventual underestimation of parasite abundance cannot be excluded, especially for *Clinostomum*, which has been reported in the skull and brain of its fish hosts (Aghlmandi et al. 2018; Shamsi et al. 2021). Parasites were recovered and preserved in either 70% or 96% ethanol for further optic and molecular identification. For scanning electron microscopic (SEM) examination, several randomly selected specimens were fixed in 2.5% glutaraldehyde (in 0.1 M sodium cacodylate buffer at pH 7.2), dehydrated through a graded ethanol series (30%, 50%, 70%, 90%, and 100%), then dried in an Emitech K850 (Quorum Technologies, Laughton, UK) critical point dryer using CO_2_. After mounting, the specimens were coated with platinum in a Q150T-ES sputter coater (Quorum Technologies, Laughton, UK) before examination with a Regulus 8230 scanning electron microscope (Hitachi, Tokyo, Japan) at an accelerating voltage of 2 kV. Parasite indices were calculated following the terminology of Bush et al. (1997).

Molecular identifications were done on randomly selected individuals in order to support the morphological determinations. DNA extraction, amplification, and Sanger sequencing were performed by Eurofins Genomics (Ebersberg, Germany). Both Nematoda and Digenea parasites were identified using the nuclear internal transcribed spacer (ITS) rDNA (ITS1, 5.8S, and ITS2) with the same primers 81_f 5′-GTAACAAGGTTTCCGTAGGTGAA-3′ (Gustinelli et al. 2010) and ITS2.S_r 5′-CCTGGTTAGTTTCTTTTCCTCCGC-3′ (Matthews and Cribb 1998), following Mazzone et al. (2019). For Nematoda, the mitochondrial cytochrome oxidase subunit 1 (COI) marker was also amplified using H7005 5′‒ACNACRTARTANGTRTCRTG‒3′ and L6625 5′‒TGRTTYTTYGGNCAYCC‒3′ primers (Hafner et al. 1994). DNA amplification was performed by PCR in a final 20 µl volume containing 10 µl of GoTAQ-HotStart Green MasterMix from Promega (Madison WI, USA), 4.5 µl of each of the two primers at 10 pM, and 1 µl of extracted DNA. After denaturation for 2 min at 95 °C, the PCR was run for 35 cycles of 1 min, 95 °C; 30 s, 50 °C; 1 min 30 s, 72 °C, before a final extension for 10 min at 72 °C and conserved at 4 °C on a Biometra Tadvanced thermocycler (Biometra GmbH, Göttingen, Germany). Forward and reverse sequences were assembled and cleaned with Geneious Prime ® 2020.2.4 (https://www.geneious.com). Obtained sequences were deposited in GenBank (accession numbers from PP888044-PP888052, PP888083-PP888092 and PP887879-PP887887), and were first blasted through the NCBI platform (https://blast.ncbi.nlm.nih.gov/) in order to have a preliminary identification. When several sequences were available in GenBank, molecular analyses were carried out. Sequences were aligned using MEGA X (Kumar et al. 2018) and the MUSCLE algorithm (Edgar 2004) with a molecular dataframe of reference from previous studies (Lazarova et al. 2006; Caffara et al. 2011; Xiong et al. 2013; Sereno-Uribe et al. 2013; Maleki et al. 2018; Li et al. 2018; Nitta and Ishikawa 2019; Locke et al. 2019; Pekmezci and Bolukbas 2021; Youssefi et al. 2023) (Supplementary data). JModeltest v.2.1.1 (Darriba et al. 2012) was used to estimate the best evolution model for the Bayesian phylogenetic inference analyses selected under the Bayesian Information Criterion (GTR + I for COI of Nematoda and HKY + G for ITS of Digenea). The percentage of divergence between sequences (p-distances) was calculated using MEGA X. The phylogenetic tree was constructed with MrBayes v.3.2.6 (Ronquist et al. 2012). Two independent analyses were run for 10 million generations, sampling every 200 generations. The convergence of the two analyses was checked and the tree obtained is a consensus with 10% of the trees discarded as burn-in. Two phylogenetic trees were then reconstructed on the ITS marker (145 sequences, 915 bp) for Digenea and COI marker (14 sequences, 385 bp) for Nematoda.

Median-joining networks were constructed for COI and ITS datasets from respectively Nematoda and Digenea using Network v.4.6 (Bandelt et al. 1999). A maximum parsimony algorithm was applied with the criterion “frequency > 1” to simplify the complex branching scheme and generate networks representing the most parsimonious relationships. Genetic diversity indices (haplotype diversity, number of polymorphic sites, and number of haplotypes) were calculated using DnaSP V6 (Rozas et al. 2017).

## Results

Following complete parasitological examination, two different parasites were recovered from the sampled *P. fluviatilis*: Nematoda were morphologically identified as larvae of *Eustrongylides* sp. (Nematoda: Dioctophymatidae) and Digenea as metacercariae of *Clinostomum* (Trematoda: Clinostomidae).

### *Eustrongylides*

**Fig. 2:**
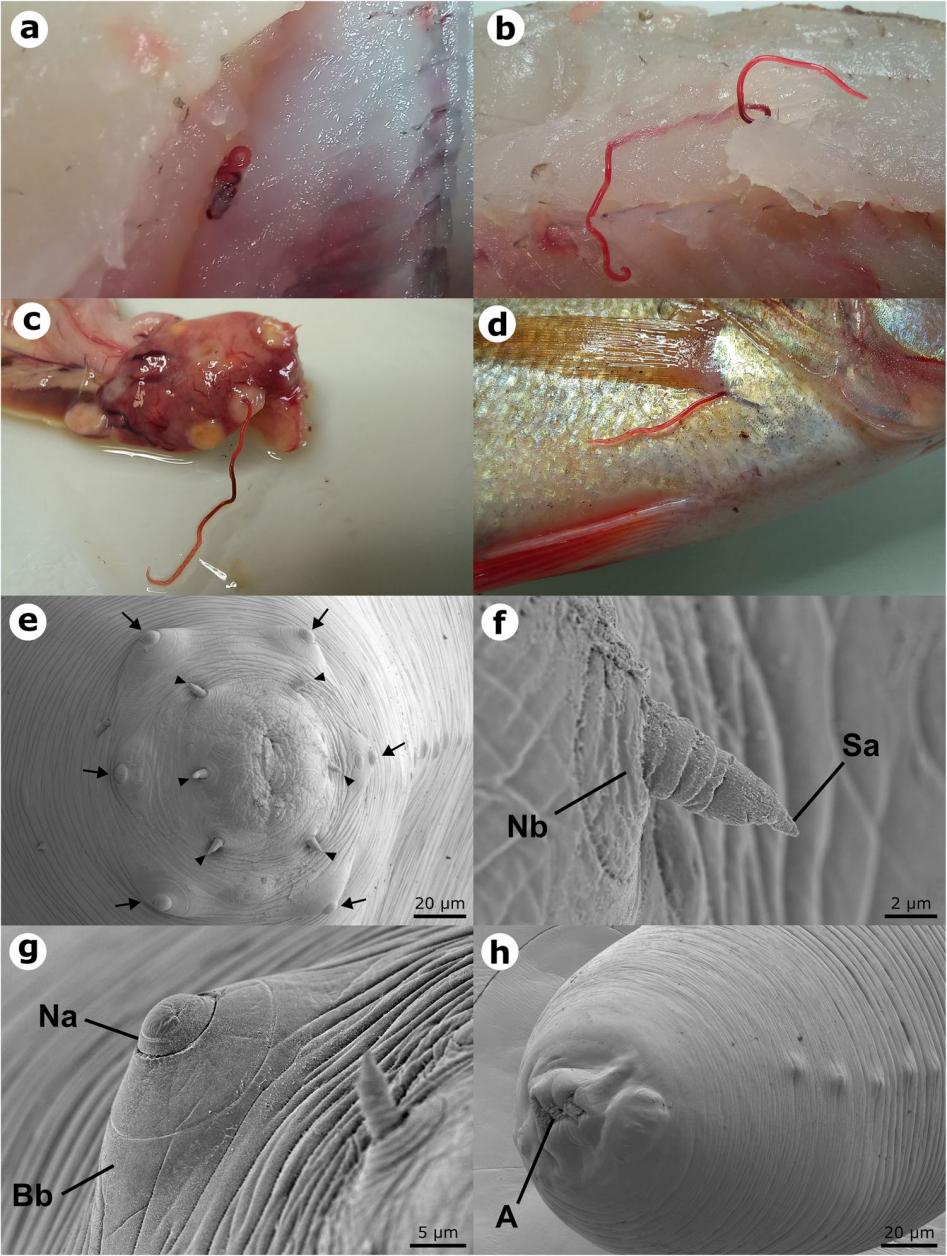
*Eustrongylides* optic and scanning electron microscopy. **a** Coiled in and **b** free and moving through *P. fluviatilis* muscle. **c** Exiting a cyst in *P. fluviatilis* liver. **d** Exiting through the skin upon its host’s death. **e** Cephalic end showing both inner (arrowheads) and outer (arrows) papillae circles and the mouth. **f** Spike-like papillae of the inner circle with narrow base (Nb) and spike-like apex (Sa). **g** Nipple-like papillae of the outer circle with broad base (Bb) and nipple-like apex (Na). **h** Posterior end showing the terminal anus (A)

*Eustrongylides* larvae were found coiled in or moving through the muscles, in the swim bladder, in the abdominal cavity, and encysted in the liver. They were bright to dark red in color (Fig. 2a, b, c, d). The individuals observed in SEM showed 12 cephalic papillae arranged in 2 circles of 6 papillae each (2 lateral, 2 subdorsal, and 2 subventral with lateral papillae slightly more anterior than subdorsal and subventral papillae, Fig. 2e). Papillae of the inner circle had narrow bases and spike-like apices (Fig. 2f) and papillae of outer circle had broad bases and nipple-like apices (Fig. 2g). The anus was terminal (Fig. 2h).

**Fig. 3:**
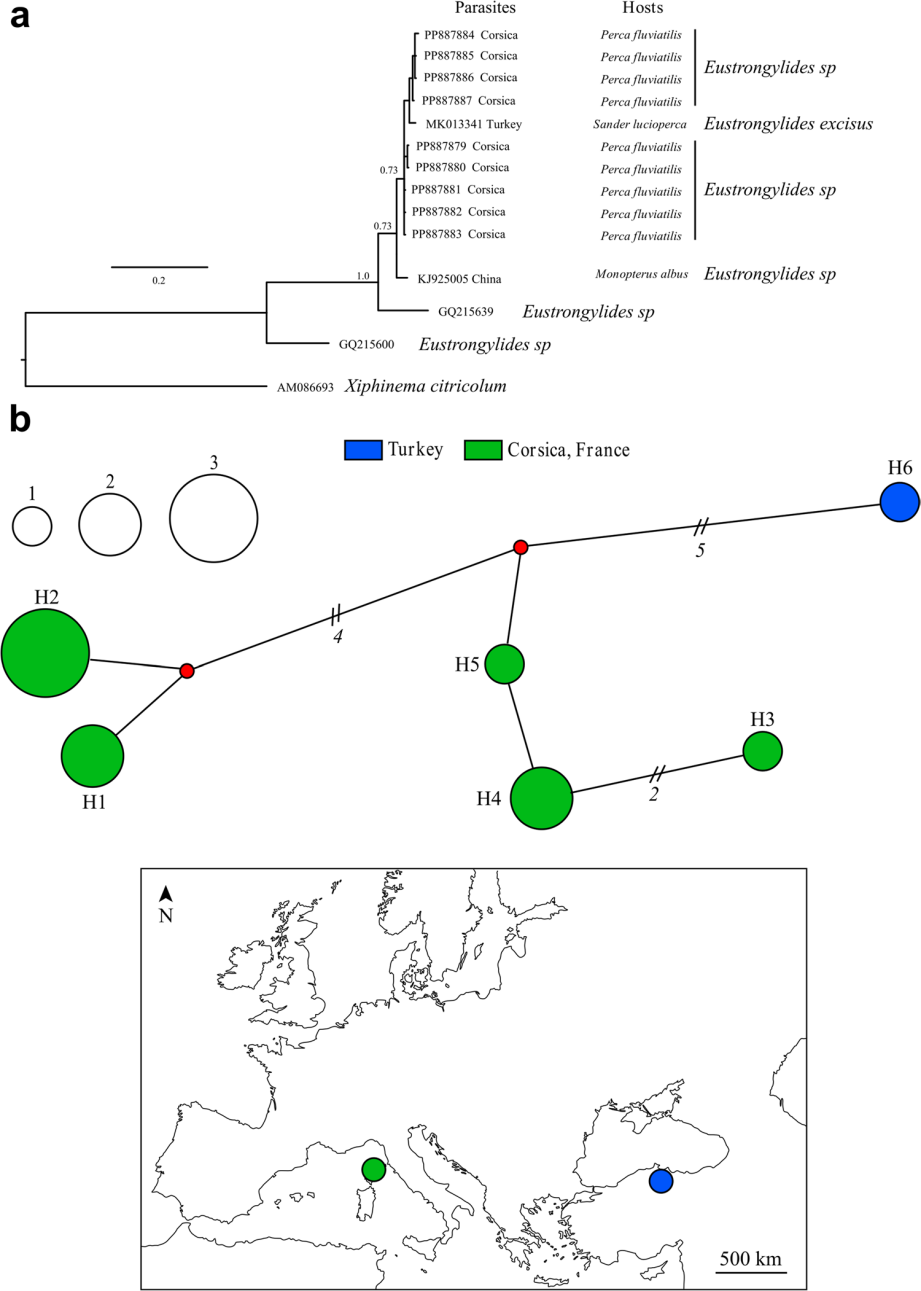
Phylogenetic tree by Bayesian inference with a GTR + I model based on COI data (385 bp) tree of *Eustrongylides*, identifying 9 specimens sampled in Padula reservoir, Corsica. Numbers at nodes correspond to posterior probability values. Corresponding host species are indicated when available. No sequences were available for *E. tubifex* (**a**). Haplotypes network obtained for *Eustrongylides*, on the 10 sequences generated in the present study and retrieved from GenBank. Circle size is proportional to the observed haplotype frequencies and red points represent hypothetical haplotypes. Colors highlight specimens’ origin (**b**)

The phylogenetic tree supported the identification of Corsican specimens as members of the genus *Eustrongylides*. However, our COI sequences obtained from *P. fluviatilis* in Corsica clustered with a specimen (GenBank accession number MK013341) recovered from a pikeperch *S. lucioperca* (Linnaeus, 1758) sampled in Derbent Dam Lake, Turkey (Pekmezci and Bolukbas 2021) and attributed to *Eustrongylides excisus* Jägerskiöld, 1909 (Fig. 3a). Our sequences are grouped in five haplotypes and two haplogroups with an average divergence of 2% (Fig. 3b). These results are also in accordance with the ITS identification using BLAST where our sequences matched at 100% with the only available sequence in GenBank with a species name (KU963206) as *E. excisus* from a Northern pike *Esox lucius* Linnaeus, 1758 from Freidoonkenar, south of the Caspian Sea (Mazandaran province, Iran) (Youssefi et al. 2023).

The observed prevalence was 90% as only the smallest *P. fluviatilis* (the only one being around 12 cm long) was found to be uninfected. The mean intensity (minimum–maximum) for *Eustrongylides* spp. was 29.6 ± 22.7 (3–64).

### *Clinostomum complanatum*

**Fig. 4:**
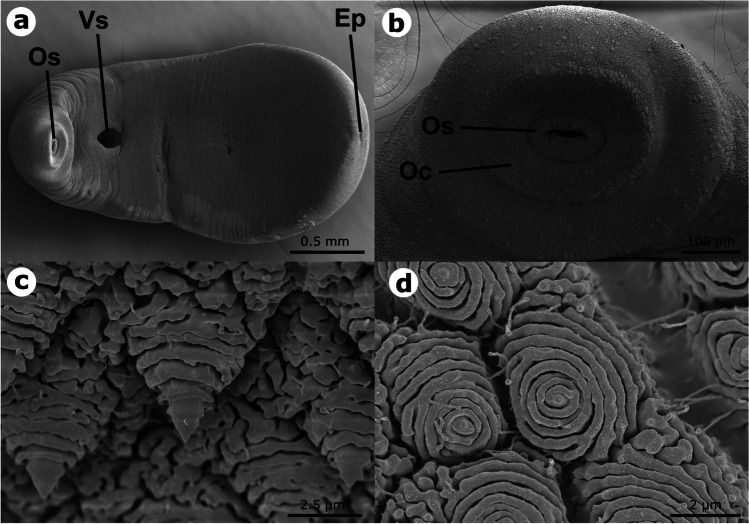
*Clinostomum complanatum* scanning electron microscopy. **a** Whole individual with the oral sucker (Os) and collar, ventral sucker (Vs), and excretory pore (Ep) clearly visible. **b** Cephalic region with the oral sucker (Os), oral collar (Oc), and sensory papillae distributed around the oral sucker. **c** Tegumental spines in the anterior region and **d** in the midbody region

Digenea metacercariae were determined as *Clinostomum* following Caffara et al. (2011). *Clinostomum* metacercariae were mainly distributed in the muscle of its hosts (75.4%) and in the opercular cavity and on gill arches (20.3%), and to a lesser extent in the abdominal cavity (2.9%) and the swimbladder (1.4%). SEM observation of excysted *Clinostomum* metacercariae showed an elongated body, wider in the gonadal region, with a terminal excretory pore (Fig. 4a). The oral sucker was small and surrounded by a prominent oral collar (Fig. 4b). The tegument was covered in spines along the body, sharper in the oral region (Fig. 4c) and more rounded midbody (Fig. 4d).

**Fig. 5:**
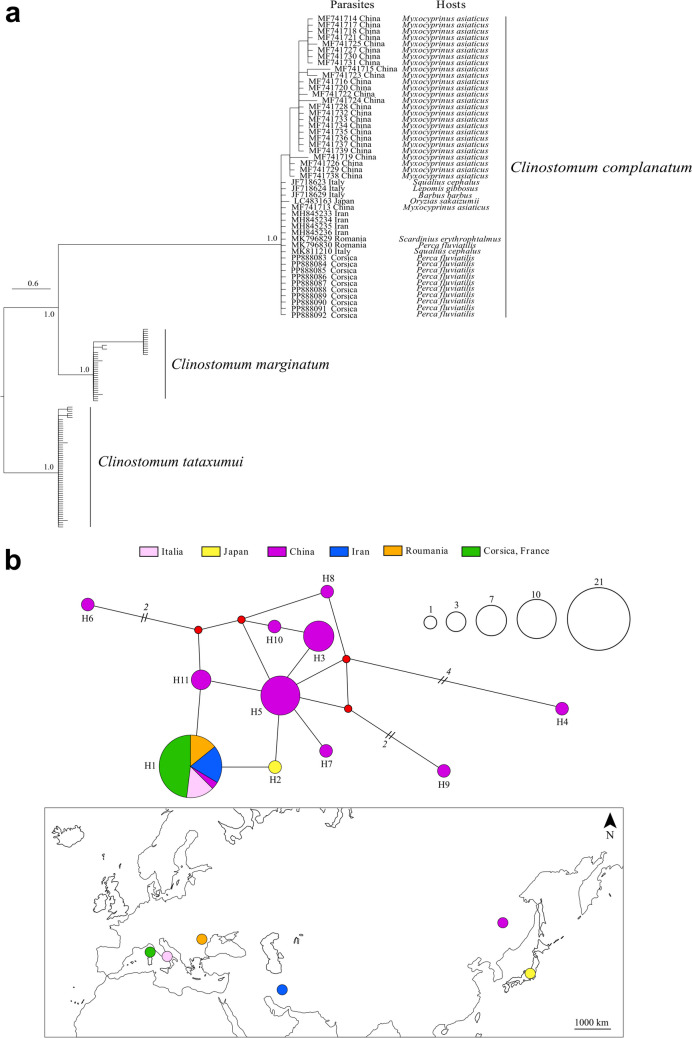
Phylogenetic tree by Bayesian inference with a HKY + G model based on ITS data (915 bp) of *Clinostomum*, identifying 10 specimens sampled in Padula reservoir, Corsica. Numbers at nodes correspond to posterior probabilities values. Corresponding host species are indicated when available (**a**). Haplotypes network obtained for *Clinostomum complanatum*, on the 48 sequences generated in the present study and retrieved from GenBank. Circle size is proportional to the observed haplotype frequencies and red points represent hypothetical haplotypes. Colors highlight specimens’ origin (**b**)

The ITS phylogenetic tree includes our sequences from Corsica within the *Clinostomum complanatum* (Rudolphi, 1814) group with an average intrinsic distance of 0.2% (Fig. 5a). The haplotype network highlights that our sequences share the same haplotype (H1) as Italian, Romanian, Iranian, and Chinese specimens (Fig. 5b) and thus seems widely distributed in Eurasia. There was a total of 11 haplotypes, with 0.752 haplotype diversity and 14 polymorphic sites.

Morphological and molecular data corroborate the identification of Digenea parasites as *C. complanatum*.

The observed prevalence was 90%. The mean intensity (minimum–maximum) was 7.7 ± 3.9 (1–11) for *C. complanatum*.

## Discussion

While molecular phylogeny enabled us to identify *C. complatanatum* with confidence, the lack of consensus between morphological and molecular data prevents a definitive species diagnosis in the case of *Eustrongylides* Nematoda. The outer circle papillae being larger than the inner circle papillae and the shape of these papillae are characteristics of *Eustrongylides tubifex* (Nitzsch in Rudolphi, 1819) according to the key to *Eustrongylides* third-stage larvae presented in Moravec (1994), while *E. excisus* shows similar dimensions for inner and outer circle papillae (Measures 1988; Moravec 1994; Gupta 2018; Mazzone et al. 2019). Ambiguity in morphological features may have led to confusion, especially for larval stages which have been reported not to display features allowing a reliable morphological species identification (Moravec and Nagasawa 2018; Mazzone et al. 2019). The non-exhaustiveness of molecular databases is a further issue encountered when studying *Eustrongylides* as the vast majority of available sequences are identified up to the genus level for these organisms. As previously noted by Shamsi et al. (2023a, b), there is no sequence available for *E. tubifex* and very few for *E. excisus*, which prevents any definite conclusion regarding the identification of the Corsican specimens. The simultaneous study of morphological features and DNA sequences of several *Eustrongylides* species could enable clarification of this situation. The grouping of the sequences obtained in the present study in two distinct haplogroups (H1 and H2 and H3, H4, and H5) hints at the hypothesis of two origins for this parasite, and possibly two distinct species. The occurrence of both *Eustrongylides* and *C. complanatum* are reported here for the first time in France and must be recorded in the French taxonomic register (TAXREF) of the INPN database (TAXREF 2024).

*Eustrongylides* prevalence in Padula reservoir (90%) is among the highest in *P. fluviatilis* in Europe, where reported prevalence ranges from 0.6% for *E. tubifex* and 13.9% for *E. excisus* in Bulgaria (Lake Srebarna) and 3.3%, 6.8%, and 10.0% for *Eustrongylides* spp. in Italy (Lake Bracciano, Trasimeno, and San Michele, respectively) to 72.0–100.0% and 94.0–100.0% for *E. excisus* in Ukraine (Zaporizhya Reservoir) and Moldova (Prut-Dniester interriveran), respectively (Branciari et al. 2016; Menconi et al. 2020b; Honcharov et al. 2022; Maggio et al. 2024). There does not seem to be a clear relation between *Eustrongylides* prevalence and the type of habitat. However, the identification of *Eustrongylides* larvae can be problematic and species-specific prevalence variations are not to be excluded.

The distribution of *C. complanatum* metacercariae mostly in the muscle and gill cavity and arches is consistent with previous reports of this parasite from fish hosts (e.g., Wang et al. 2017; Menconi et al. 2020a). The prevalence of infection was higher than that reported from Italy (14.3–21.4%, Lake Endine), Turkey (13.1% and 53.8%, Lake Sığırcı and Gala, respectively), and Czech Republic (15.0%, Morava river basin) (Kadlec et al. 2003; Çolak 2013; Soylu 2014), as well as infection intensities. As already noted by Menconi et al. (2020a), the higher prevalence observed by Soylu (2014) in Lake Gala, compared to other localities, may be attributed to its position on the avian migration route, and thus the attraction of numerous final hosts. In the same way, numerous piscivorous birds may be attracted to Padula reservoir by the nearby Biguglia lagoon, a RAMSAR wetland situated on a bird migration route (MTES 2020).

### Occurrence of intermediate and final hosts

Numerous species of aquatic Oligochaeta, the usual first intermediate hosts of *Eustrongylides* spp., are present in Corsica (Orsini 2008). A relatively wide range of intermediate piscine hosts is known to be suitable for *Eustrongylides* in Europe, most notably predatory species e.g. *P. fluviatilis*, *S. lucioperca*, and the European catfish *Silurus glanis* Linnaeus, 1758, in Eastern Europe and more recently from Southern Europe (Italy) (Shukerova et al. 2010; Bjelić-Čabrilo et al. 2013; Branciari et al. 2016; Goncharov et al. 2018; Menconi et al. 2020b; Honcharov 2020; Guardone et al. 2021; Honcharov et al. 2022). Numerous piscivorous birds are reported as final hosts for *Eustrongylides* Nematoda, i.e., great cormorants *Phalacrocorax carbo* (Linnaeus, 1758), Anatidae (wild ducks) as well as various Ciconiformes, e.g., *Egretta thula* (Molina, 1803) (e.g., Honcharov et al. 2022). Moreover, Spalding and Forrester (1993) also report the occurrence of *Eustrongylides* from the great blue heron *Ardea herodias* Linnaeus, 1758. Such species, or at least congenerics, are known to occur in Corsica, where *P. carbo*, *Ardea cinerea* Linnaeus, 1758, *Ardea purpurea* Linnaeus, 1766, *Egretta garzetta* (Linnaeus, 1766) and the Anatidae *Anas platyrhynchos* Linnaeus, 1758, and *Netta rufina* (Pallas, 1773) are known to occur in the island freshwaters (Collective Losange 2017) (Table 1).

*Clinostomum complanatum* has been reported from several Lymnaeidae (Gastropoda) species pertaining to various genera, such as *Radix euphratica* (Mousson, 1874), *Radix plicatula* (W. H. Benson, 1842), *Ampullaceana balthica* (Linnaeus, 1758), *Lymnaea stagnalis* (Linnaeus, 1758) and *Bullastra lessoni* (Deshayes, 1831) (Gibson 2019; Nazarbeigy et al. 2021; Shamsi et al. 2023a, b; Wang et al. 2017). Even though there are no available data concerning malacological communities of Padula reservoir, several Lymnaeidae have been reported from Corsica: *Stagnicola palustris* (O. F. Müller, 1774), *Galba truncatula* (O. F. Müller, 1774), *Peregriana peregra* (O. F. Müller, 1774) and *Pseudosuccinea columella* (Say, 1817) (Alba et al. 2023; Dominici et al. 1996; Orsini 2008). It is thus likely that *C. complanatum* uses of these Lymnaeidae species as a first intermediate host in Corsica. It would be of interest to carry out a malacological survey in order to determine whether the larval stage of *C. complanatum* is present in the freshwater snails of the Padula reservoir. In Europe, *C. complanatum* is reported from several fish hosts, from numerous countries along the Danube river basin (Czechia, Hungary, Romania, Serbia, Slovakia, and Ukraine) and from Italy (Gaglio et al. 2016; Fedorčák et al. 2019; Menconi et al. 2020b). *Clinostomum complanatum* uses piscivorous birds final hosts either reported from Corsica or taxonomically close to hosts present on the island as its occurrence is noted in *Ardea alba* Linnaeus, 1758, *A. purpurea*, *E. garzetta*, *A. platyrhynchos*, *P. carbo*, and *Larus* gulls (Shamsi et al. 2013; El-Dakhly et al. 2018; Gibson 2019; Nazarbeigy et al. 2021) (Table 1). All the hosts needed to complete the life cycle of both *Eustrongylides* sp. and *C. complanatum* are therefore present in Corsica, and the introduction to the island of several piscine species most likely favors both these parasites’ life cycle.

As Corsica is a Mediterranean island situated on bird migration routes (Bruderer and Liechti 1999; Jourdain et al. 2007; Maggini et al. 2020), the possibility that *Eustrongylides* sp. and *C. complanatum* may have been transported with migrating birds before finding suitable fish hosts rather than being co-introduced with piscine hosts cannot be ruled out. It has been reported, for example, that *Phalacrocorax carbo* has possibly played a key role in the recent expansion of *Eustrongylides* sp. in Central and Northern Italy (Castiglione et al. 2023). However, the hypothesis of co-introduction through infected fish is also probable. Further investigations are needed to resolve this issue, if indeed it can be resolved. Furthermore, the possibility of an introduction via the first intermediate hosts cannot be ruled out. However, the lack of data concerning eventual introductions of Lymnaeidae and Oligochaeta in Corsica prevents from properly assessing this possibility.

Additionally, infection with *Clinostomum* or *Eustrongylides* were recorded in numerous amphibian species (e.g. Yildirimhan et al. 2012; Caffara et al. 2014; León-Règagnon 2019). Seven amphibian species are present in Corsica, among which three endemics. A perspective would be to conduct a parasitological examination of these organisms in order to determine if *Clinostomum complanatum* and/or *Eustrongylides* sp. could have been transferred to local amphibians. Indeed, it would be of interest as these organisms may constitute an additional reservoir for these zoonotic parasites.

### Risk of food-borne Eustrongylidosis and Clinostomiasis in human

Both parasites are zoonotic as several human cases have been reported, mostly in eastern Asia (Japan, Korea) for *Clinostomum* and in North America (USA) for *Eustrongylides* (*e.g.*, Gunby 1982; Wittner et al. 1989; Isobe et al. 1994; Chung et al. 1995; Narr et al. 1996; Hara et al. 2014; Kim et al. 2019). The infection route in human for both parasites is the consumption of either live or raw fish: *Eustrongylides* infections were caused either by the consumption of live bait by recreational anglers or sashimi made from unfrozen fresh fish (Gunby 1982; Wittner et al. 1989; Narr et al. 1996) and *C. complanatum* infections by the consumption of raw freshwater or brackish water fish (Isobe et al. 1994; Chung et al. 1995; Hara et al. 2014; Kim et al. 2019). Given the capacity of both *C. complanatum* and *Eustrongylides* spp. to infect humans, we recommend that the occurrence of these parasites in the fish of Padula reservoir should be monitored, as prevalence can change over time as was shown in Trasimeno lake, Italy (Franceschini et al. 2022). The cccurrence of the two parasites could only be analyzed in *P. fluviatilis* but Nematoda matching the description of *Eustrongylides* were also reported by a recreational angler from *S. lucioperca* but not from *M. salmoides*, both species occurring in Padula reservoir. *Perca fluviatilis* and *S. lucioperca* are prized by fishermen for their tasty meat and are commonly consumed (Fleury and Le Mesle 2022). Both the local population and tourists should be warned to avoid the raw consumption of both *P. fluviatilis* and *S. lucioperca*. In addition, the monitoring should be extended to the other reservoirs where these fish are known to occur. The occurrence of final bird hosts is another component that should be taken into account while monitoring both parasites as the population growth of the *P. carbo* is believed to have had a major impact on the apparent expansion of *Eustrongylides* sp. in Italy (Castiglione et al. 2023).

## Conclusion

The parasites of the non-native *P. fluviatilis* were studied for the first time in the French Mediterranean island of Corsica, following the report of a recreational angler. This led to the first observation of two zoonotic parasites, the Nematoda *Eustrongylides* sp. and the Digenea *C. complanatum*. The present study is thus an example of how crucial it is that academic research and citizens communicate and work together. *Clinostomum complanatum* was reliably identified through the use of molecular and morphological study. *Eustrongylides* could not be identified at the species level, mainly because molecular databases do not cover all of this genus’ species. It is highly likely that the life cycle of both *C. complanatum* and *Eustrongylides* can be completed in Corsica as intermediate and final hots seem to be available on the island. The past introductions of numerous non-native species in Corsica may facilitate the completion of these life cycles as well as the maintenance of these parasites in Corsica. The occurrence of *C. complanatum* and *Eustrongylides* sp. is concerning from both a veterinary and human health perspective as these parasites can use a wide range of amphibians as intermediate hosts and can be acquired in humans through the consumption of raw or undercooked fish. Even if the consumption of freshly caught wild fish sushi or sashimi is not reported to be a common practice in Corsica, recreational anglers eating their catch and medical practitioners should remain vigilant. Because they potentially interact with numerous components of their ecosystem, in addition to being zoonotic, both *Eustrongylides* and *C. complanatum* are an illustration of how interconnected are human, animal, and ecosystem health, as described in the One Health framework.

### Supplementary Information

Below is the link to the electronic supplementary material.

Supplementary file1 (XLSX 18.4 KB)

## Data Availability

The DNA sequences generated during this study have been deposited in GenBank under the accession numbers PP888044-PP888052, PP888083-PP888092 and PP887879-PP887887.
